# Effects of Substance P and Neurokinin A on the Contractile Activity of Inflamed Porcine Uterus

**DOI:** 10.3390/ijms232113184

**Published:** 2022-10-29

**Authors:** Marta Brzozowska, Marta Romaniewicz, Jarosław Całka, Barbara Jana

**Affiliations:** 1 Department of Clinical Physiology, Faculty of Veterinary Medicine, University of Warmia and Mazury, Oczapowskiego 13, 10-718 Olsztyn, Poland; 2 Institute of Animal Reproduction and Food Research of the Polish Academy of Sciences, Tuwima 10, 10-748 Olsztyn, Poland

**Keywords:** endometritis, uterine contractility, tachykinins, tachykinin receptors, sows

## Abstract

Disturbances in uterine contractile activity contribute to the development of inflammation, and recent evidence indicates that tachykinins, including substance P (SP) and neurokinin A (NKA), are involved in controlling uterine function. Here, we determined the effect of *Escherichia coli* (*E. coli)*-induced inflammation on expression of protein receptor subtypes for substance P (NK1R) and neurokinin A (NK2R) in the pig myometrium as well as their role in contractility of inflamed uterus. The severe acute endometritis developed in the *E. coli* group and the expression of NK1R and NK2R proteins increased in the myometrium. Compared to the pre-administration period, SP (10^−6^ M) reduced the amplitude and frequency in the myometrium of the *E. coli* group and the amplitude was higher and the frequency was lower versus other groups. NKA reduced the amplitude and increased the frequency in endometrium/myometrium of the *E. coli* group. In this group, the amplitude was lower and the frequency was higher than in the CON and SAL groups. Our research showed that NK2R (10^−6^ M) antagonist application abolished the NKA inhibitory effect on uterine amplitude. The application of the NK1R (10^−5^ M) antagonist together with SP revealed that the inhibitory effect of SP on uterine contractility is achieved independently of the NKR1. Additionally, taking into account the fact that NKA shows an inhibitory effect with the use of NK2R on uterine amplitude suggests the possibility of therapeutic use of the antagonist as a drug increasing uterine contractility in inflammation.

## 1. Introduction

The uterus, especially endometrium, plays a key role in normal reproductive cycles as well as in placentation, embryo implantation and maintenance of pregnancy. Unfortunately, uterine bacterial infections are common diseases in both humans and animals and reproductive complications contribute to infertility and abortion. Bacterial infections in pigs caused, i.a. by *Escherichia coli* (*E. coli*), *Streptococcus* sp. and *Staphylococcus* sp., are the main cause of uterine inflammatory lesions [1].

It is well known that disorders in immune response and/or contractions can contribute to uterine inflammation. In addition, labor complications, e.g., fetal membrane retention contribute to the development of inflammatory changes [2]. Repeatedly, impaired contractility of the uterus with inflammation contributes to the presence of inflammatory exudate in the uterine cavity. During uterine inflammation, there is a significant increase in the synthesis of pro- and anti-inflammatory cytokines [3,4,5] as well as prostaglandins (PG)F2α, PGE2 and PGI2 [6,7]. It should be noted that PGF2α, PGE2, PGI2 [7,8] and leukotrienes (C4, D4) [9] have been shown to be involved in contractility of porcine uterus with inflammation.

It is established that the physiological activity of the uterus is regulated by sympathetic, parasympathetic and sensory neurons [10]. Although both cholinergic and adrenergic components are primarily involved in controlling uterine function, other neurotransmitters have also been shown to be responsible for modulating uterine contractility. To date, the role of noradrenaline, acetylcholine (ACh), somatostatin, neuropeptide Y and galanin in the contractile regulation of the porcine uterus with the bacterially induced inflammation has been described [11,12,13,14].

In the literature, there are many data concerning the role of tachykinins (TKs) in the regulation of uterine function [15]. TKs are a family of peptides that include, among others, substance P (SP) and neurokinin A (NKA). Peptides exert their effects through G protein-coupled receptors [16]. To date, three different TK receptors have been described, namely the NK1 receptor (NK1R), the NK2 receptor (NK2R) and the NK3 receptor. It has been found that endogenous TKs have a different affinity for each of the TK receptors. Accordingly, NK1R is activated preferentially by SP and NK2R by NKA [17]. It is worth noting that in the peripheral nervous system, only the SP and NKA play a role as neurotransmitters and/or neuromodulators [18]. TKs are responsible for the modulation of reproductive functions in the central and peripheral levels. The expression of SP and NKA in the hypothalamus and pituitary has been described where they affect the level of gonadotropins and, consequently, gonadal steroids. The TKs have been also found in non-neuronal cells of the ovary, uterus and placenta, as well as in the sensory nerves supplying the reproductive organs [19]. It should be mentioned that TK receptor-selective agonists are involved in the regulation of uterine smooth muscle contractility [15]. Additionally, it has been shown that TKs level in the uterus undergoes changes during pregnancy and estrous cycle [20]. Accordingly, it is likely that SP and NKA and their receptors may modulate uterine contractility during inflammation, especially considering the fact that TKs stimulate inflammatory cells [18].

For many years, pigs have been used as a research model due to similar anatomy and physiological processes to humans [21]. In relation to the present study, it was hypothesized that endometritis affects the myometrial expression of NK1R and NK2R and the participation NK1R/SP and NK2R/NKA systems in the contractile function of an inflamed uterus. Therefore, the impact of endometritis on (1) NK1R and NK2R protein expression in myometrium and (2) the role of these receptors in SP- and NKA-influenced contractile amplitude and frequency of inflamed porcine uterus was investigated.

## 2. Results

### 2.1. Expression of NK1R and NK2R Proteins

**Figure 1:**
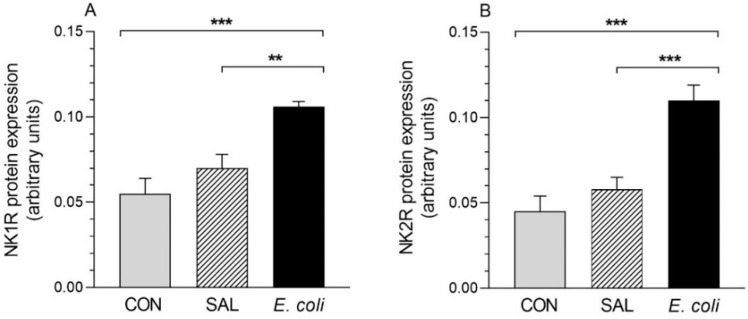
The protein expression of NK1R (**A**) and NK2R (**B**) in the porcine myometrium from the control (CON), saline (SAL) and *E. coli* groups, estimated by Western blot analysis. Blots with representative bands for each group are shown in Supplementary Figure S1. Protein levels of studied receptors are expressed as the mean ± SEM of ratios of glyceraldehyde-3-phosphate dehydrogenase (GAPDH). ** *p* < 0.01, *** *p* < 0.001 compared between groups for the same type of receptor.

The levels of NK1R and NK2R proteins in the myometrial tissues of the *E. coli* group were significantly higher compared to the CON and SAL groups (Figure 1). The protein expression of these receptors in the CON and SAL groups did not differ significantly. Protein bands in the porcine myometrium pointed the molecular mass for NK1R and NK2R (Figure S1). NK1R and NK2R proteins coincided with a 46 kDa molecular weight marker. Western blot analysis performed using mouse and porcine duodenal tissues showed bands at 46 kDa and 46 kDa that were recognized as NK1R and NK2R proteins analogously (positive control) (Figure S2). It should be mentioned that the bands were not recognized after omitting primary antibodies (negative control).

### 2.2. Spatial Distribution of NK1R and NK2R

**Figure 2:**
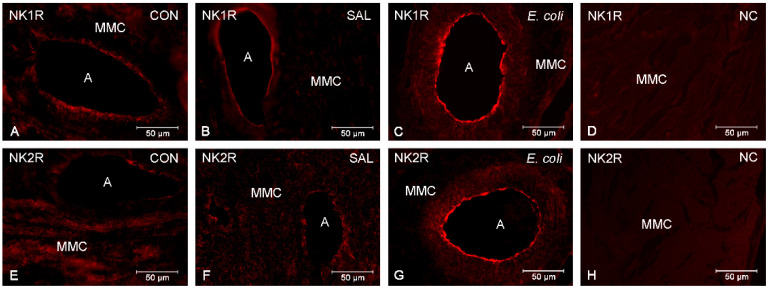
Representative pictures of NK1R (**A**–**C**) and NK2R (**E**–**G**) in the myometrium of gilts from the control (CON), saline (SAL) and *E. coli* (*E. coli*) groups. Positive immunoreaction to NK1R is visible in myometrial muscular cells (MCM) and arteries (**A**) of the control (**A**), saline-(**B**) and pathogens (**C**)-injected uteri. Expression of NK2R display MCM and A in the control (**E**), saline-injected (**F**) and inflamed (**G**) uteri. Negative control (NC) for NK1R (**D**) and NK2R (**H**) was obtained following the use of rabbit normal IgG instead of primary antibodies.

Immunofluorescence staining revealed the expression of NK1R and NK2R in the endothelium and muscle layer of blood vessels as well as in the muscle cells of myometrium of the CON (Figure 2A,E, respectively), SAL (Figure 2B,F, respectively) and *E. coli* (Figure 2C,G, respectively) groups. The expression of these receptors has been also noted in the porcine duodenum (positive control) (Figure S3). It was noted that the use of normal rabbit IgG in place of the primary antibodies did not result in labeling of NK1R (Figure 2D) and NK2R (Figure 2H).

### 2.3. Influence of SP on Myometrial Contractility

#### 2.3.1. Comparison of the SP Influence to the Period before Its Application

**Figure 3:**
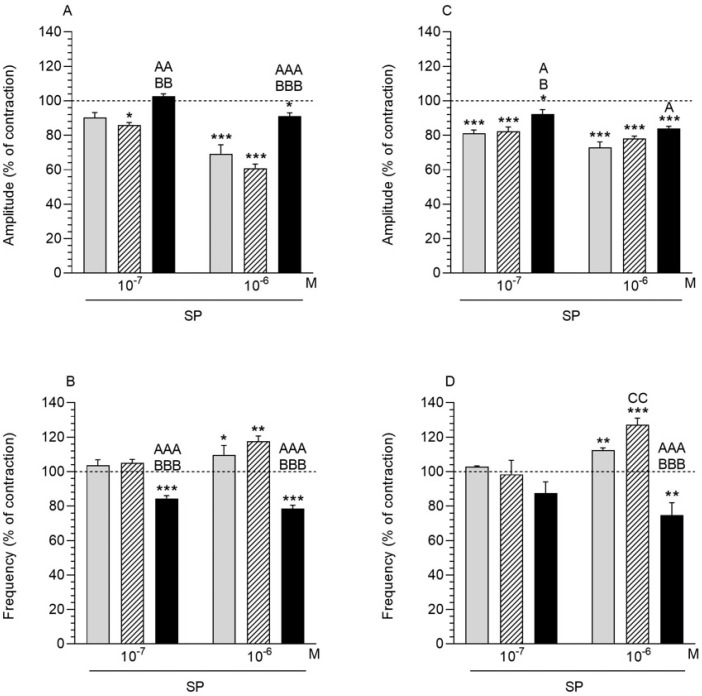
The effect of substance P (SP) on the contractile amplitude (**A**,**C**) and frequency (**B**,**D**) in the myometrium (**A**,**B**) and endometrium/myometrium (**C**,**D**) strips of gilts from the CON (grey bars), SAL (hatched bars) and *E. coli* (black bars) groups. Results were obtained from five pigs in each group. The effects of individual SP doses are depicted as percentages (mean ± SEM) of the baseline (pretreatment period) contractile amplitude and frequency, taken as 100% (horizontal lines). * *p* < 0.05, ** *p* < 0.01, *** *p* < 0.001 compared to the basal value in each group; ^A^ *p* < 0.05, ^AA^ *p* < 0.01, ^AAA^ *p* < 0.001 compared between the CON and *E. coli* groups for the same treatment; ^B^ *p* < 0.05, ^BB^ *p* < 0.01, ^BBB^ *p* < 0.001 compared between the SAL and *E. coli* groups for the same treatment; ^CC^ *p* < 0.01 compared between the CON and SAL groups for the same treatment.

After the SP application at a dose of 10^−7^ M, the amplitude in the myometrium significantly decreased in the SAL group (Figure 3A). The significant reduction in the myometrial amplitude was observed in all the studied groups at a 10^−6^ M dose. In the case of the myometrium frequency, both in the CON and SAL groups, a significant increase in this parameter was recorded with the use of SP at a concentration of 10^−6^ M (Figure 3B). In the *E. coli* group, the administration of SP at both doses resulted in a significant decrease in frequency.

#### 2.3.2. Comparison of the SP Influence between Groups

The myometrial amplitude in the *E. coli* group was significantly increased compared to the other groups after SP administration at both doses (Figure 3A). An inverse relationship was observed for the frequency, where a significant decrease in this parameter was visible in the *E. coli* group compared to the other studied groups (Figure 3B).

### 2.4. SP Influence on the Endometrium/Myometrium Contractility

#### 2.4.1. Comparison of the SP Influence to the Period before Its Application

In the CON, SAL and *E. coli* groups, a significant reduction in amplitude in the endometrium/myometrium was recorded after SP administration at doses of 10^−7^ and 10^−6^ M (Figure 3C). In the case of frequency, SP application at a dose of 10^−6^ M significantly increased the parameter in the CON and SAL groups, while the opposite effect was noted in the *E. coli* group (Figure 3D).

#### 2.4.2. Comparison of the SP Influence between Groups

In response to SP at a dose of 10^−7^ M, the amplitude in endometrium/myometrium of the *E. coli* group was significantly enhanced compared to other groups (Figure 3C). In turn, the administration of SP at a dose of 10^−6^ M caused a significant increase in this parameter in the *E. coli* group compared to the CON group. After using SP at a dose of 10^−6^ the frequency in the *E. coli* group was significantly lower than in other groups (Figure 3D). In the endometrium/myometrium of the SAL group this parameter was significantly increased compared in the CON group.

### 2.5. NKR1 Antagonist and SP Influence on the Myometrium Contractility

#### 2.5.1. Comparison of the Antagonist and SP Influence to the Period before Their Application

**Figure 4:**
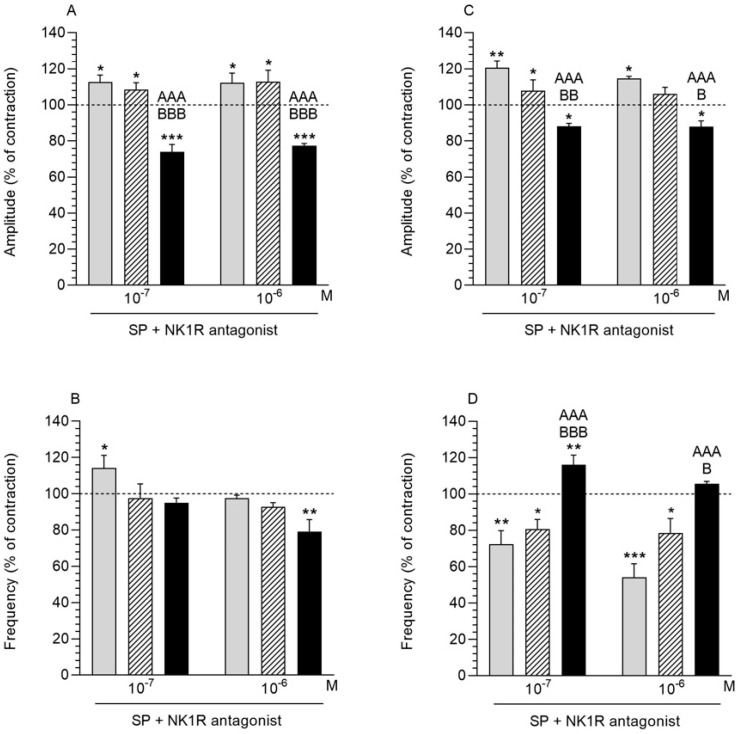
The influence of substance P (SP) on the contractile amplitude (**A**,**C**) and frequency (**B**,**D**) in the myometrium (**A**,**B**) and endometrium/myometrium (**C**,**D**) strips of gilts from the CON (grey bars), SAL (hatched bars) and *E. coli* (black bars) groups after using NK1R antagonist. Results were calculated for five gilts in each group. The effects of antagonist and individual SP doses are presented as percentages (mean ± SEM) of the baseline (pretreatment period) contractile amplitude and frequency, taken as 100% (horizontal lines). * *p* < 0.05, ** *p* < 0.01, *** *p* < 0.001 compared to the basal value in each group; ^AAA^ *p* < 0.001 compared between the CON and *E. coli* groups for the same treatment; ^B^ *p* < 0.05, ^BB^ *p* < 0.01, ^BBB^ *p* < 0.001 compared between the SAL and *E. coli* groups for the same treatment.

After using the NK1R antagonist (10^−5^ M) with SP (10^−7^, 10^−6^ M) in the CON and SAL groups, the amplitude in the myometrium significantly increased (Figure 4A). In turn, the antagonist and both doses of SP caused a significant decrease in the amplitude in the *E. coli* group. In the CON group, administration of the antagonist and SP at a dose of 10^−7^ M led to a significant increase in the frequency of myometrium (Figure 4B). The myometrium of *E. coli* groups responded with a significant decrease in the frequency following the exposure to both antagonist and SP (10^−6^ M).

#### 2.5.2. Comparison of the Antagonist and SP Influence between Groups

The myometrial amplitude in the *E. coli* group under the influence of NK1R antagonist (10^−5^ M) with SP (10^−7^, 10^−6^ M) was significantly lower compared to other groups (Figure 4A). The effect of the antagonist and SP on the myometrial frequency did not differ significantly between the studied groups (Figure 4B).

### 2.6. NK1R Antagonist and SP Influence on the Endometrium/Myometrium Contractility

#### 2.6.1. Comparison of the Antagonist and SP Influence to the Period before Their Application

The administration of the NK1R antagonist (10^−5^ M) together with SP (10^−6^, 10^−7^ M) caused a significant increase in the amplitude of the endometrium/myometrium in the CON group and a significant decrease in the parameter in the *E. coli* group (Figure 4C). In the SAL group, the antagonist and SP only at a dose of 10^−7^ M caused a significant increase in amplitude. Recording of the endometrium/myometrium frequency revealed a significant decrease in the parameter in the CON and SAL groups after the combined administration of the antagonist and SP at both doses (Figure 4D). In the endometrium/myometrium of *E. coli* group, a significant increase in the frequency was observed after administration of the antagonist together with SP at a concentration of 10^−7^ M.

#### 2.6.2. Comparison of the Antagonist and SP Influence between Groups

In the endometrium/myometrium of the *E. coli* group, the NK1R antagonist (10^−5^ M) and SP (10^−6^, 10^−7^ M) led to a significant decrease in the amplitude (Figure 4C) and a significant increase in the frequency (Figure 4D) compared to the CON and SAL groups.

### 2.7. NKA Influence on the Myometrium Contractility

#### 2.7.1. Comparison of the NKA Influence to the Period before Its Application

**Figure 5:**
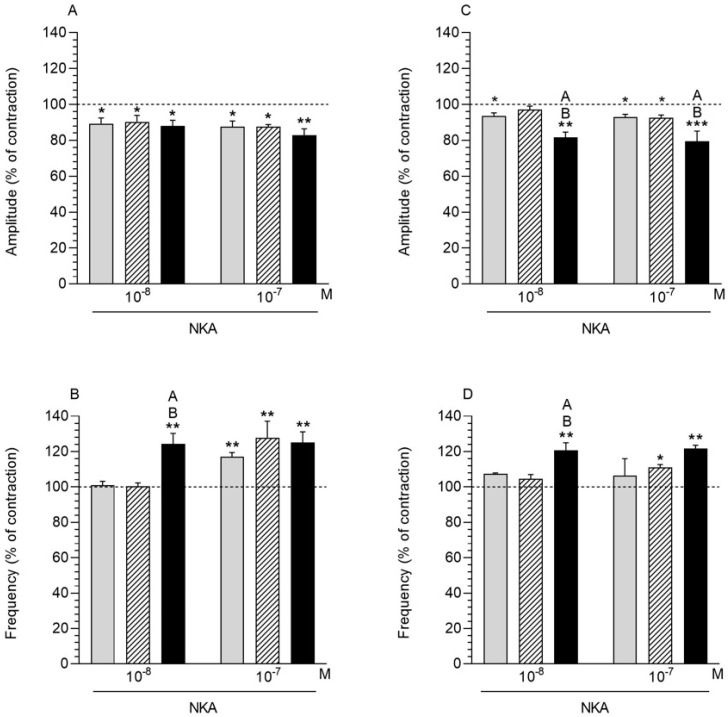
The effect of neurokinin A (NKA) on the contractile amplitude (**A**,**C**) and frequency (**B**,**D**) in the myometrium (**A**,**B**) and endometrium/myometrium (**C**,**D**) strips of gilts from the CON (grey bars), SAL (hatched bars) and *E. coli* (black bars) groups. Results were calculated for five gilts in each group. The effects of individual NKA doses are depicted as percentages (mean ± SEM) of the baseline (pretreatment period) contractile amplitude and frequency, taken as 100% (horizontal lines). * *p* < 0.05, ** *p* < 0.01, *** *p* < 0.001, compared to the basal value in each group; ^A^ *p* < 0.05 compared between the CON and *E. coli* groups for the same treatment; ^B^ *p* < 0.05 compared between the SAL and *E. coli* groups for the same treatment.

The incubation of myometrial tissue with NKA (10^−8^, 10^−7^ M) caused a significant decrease in the amplitude in all studied groups (Figure 5A). In the *E. coli* group myometrium, the NKA (10^−8^, 10^−7^ M) application resulted in a significantly increased frequency (Figure 5B). In the CON and SAL groups, the administration of NKA at a dose of 10^−7^ M caused a significant increase in the myometrial frequency.

#### 2.7.2. Comparison of the NKA Influence between Groups

The administration of NKA (10^−8^, 10^−7^ M) did not reveal significant changes in myometrial amplitude between the studied groups (Figure 5A). In turn, it was noted that the application of NKA (10^−8^ M) induced a significant increase in the frequency of the *E. coli* group myometrium compared to other experimental groups (Figure 5B).

### 2.8. NKA Influence on the Endometrium/Myometrium Contractility

#### 2.8.1. Comparison of the NKA Influence to the Period before Its Application

After the NKA application (10^−8^, 10^−7^ M), a significantly decreased amplitude of the endometrium/myometrium was visible in the CON and *E. coli* groups (Figure 5C). In the case of the SAL group, the administration of NKA only at a dose of 10^−7^ M resulted in a significant decrease in the amplitude. The study to determine the endometrium/myometrium frequency showed a significant increase in this parameter in the *E. coli* group following the NKA application at both doses (Figure 5D). Administration of NKA (10^−7^ M) resulted in a significant increase in the endometrium/myometrium frequency in the SAL group.

#### 2.8.2. Comparison of the NKA Influence between Groups

In the case of the amplitude, the administration of NKA at doses of 10^−8^ and 10^−7^ M caused a significant decrease in the *E. coli* group compared to the CON and SAL groups (Figure 5C). Administration of NKA (10^−8^ M) resulted in a significant increase in the frequency in the *E. coli* group compared to other groups (Figure 5D).

### 2.9. NK2R Antagonist and NKA Influence on the Myometrium Contractility

#### 2.9.1. Comparison of the Antagonist and NKA Influence to the Period before Their Application

**Figure 6:**
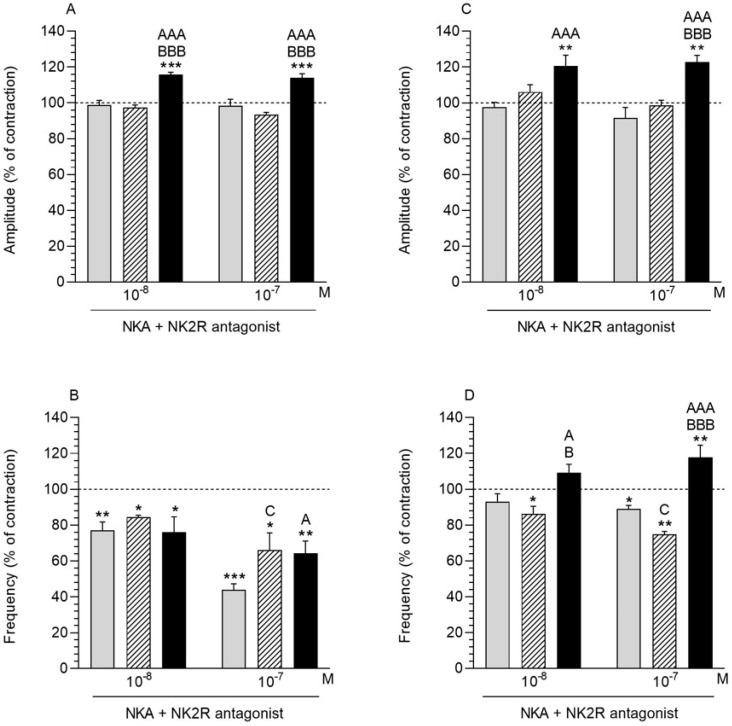
The influence of neurokinin A (NKA) on the contractile amplitude (**A**,**C**) and frequency (**B**,**D**) in the myometrium (**A**,**B**) and endometrium/myometrium (**C**,**D**) strips of gilts from the CON (grey bars), SAL (hatched bars), and *E. coli* (black bars) groups after using NK2R antagonist. Results were calculated for five gilts in each group. The effects of antagonist and individual NKA doses are presented as percentages (mean ± SEM) of the baseline (pretreatment period) contractile amplitude and frequency, taken as 100% (horizontal lines). * *p* < 0.05, ** *p* < 0.01, *** *p* < 0.001 compared to the basal value in each group; ^A^ *p* < 0.05, ^AAA^ *p* < 0.001 compared between the CON and *E. coli* groups for the same treatment; ^B^ *p* < 0.05, ^BBB^ *p* < 0.001 compared between the SAL and *E. coli* groups for the same treatment; ^C^ *p* < 0.05 compared between the CON and SAL groups for the same treatment.

NK2R antagonist (10^−6^ M) and NKA (10^−8^, 10^−7^ M) led to a significant increase in amplitude in the *E. coli* group (Figure 6A). Interestingly, the application of the antagonist of NK2R with NKA (at both doses) led to a significant decrease in the myometrial frequency in all experimental groups (Figure 6B).

#### 2.9.2. Comparison of the Antagonist and NKA Influence between Groups

The administration of the antagonist NK2R (10^−6^ M) with NKA (10^−8^, 10^−7^ M) resulted in a significant increase in the amplitude of the *E. coli* group compared to the CON and SAL groups (Figure 6A). In the case of frequency, a significant increase in the parameter was observed in the SAL and *E. coli* groups in relation to the CON group in response to the antagonist and NKA at a dose of 10^−7^ M (Figure 6B).

### 2.10. NK2R Antagonist and NKA Influence on the Endometrium/Myometrium Contractility

#### 2.10.1. Comparison of the Antagonist and NKA Influence to the Period before Their Application

The application of NK2R antagonist (10^−6^ M) together with NKA (10^−8^, 10^−7^ M) showed a significant increase amplitude in the endometrium/myometrium of *E. coli* group (Figure 6C). The frequency significantly decreased after administration of the antagonist and NKA (10^−8^, 10^−7^ M) in the SAL group and in the CON group in response to the antagonist and NKA at a dose of 10^−7^ M (Figure 6D). The administration of the antagonist and NKA at a dose of 10^−7^ M caused a significant increase in the frequency in the *E. coli* group.

#### 2.10.2. Comparison of the Antagonist and NKA Influence between Groups

The amplitude in the *E. coli* group was significantly higher compared to the CON and SAL groups after administration of NK2R antagonist (10^−6^ M) and NKA at a dose of 10^−7^ M (Figure 6C). The increase in amplitude in the *E. coli* group resulting from the application of the NKA antagonist at a dose of 10^−8^ M was significant only in relation to the CON group. It was observed that the antagonist of NK2R together with NKA (10^−8^, 10^−7^ M) induced a significant increase in the frequency in *E. coli* group compared to other groups (Figure 6D). Only the application of the antagonist with NKA at a dose of 10^−7^ M resulted in a significant decrease in the frequency in SAL group compared to the CON group.

## 3. Discussion

The presented research describes the effect of bacterial endometritis on the expression of NK1R and NK2R proteins in the myometrium as well as the participation of SP, NKA, NK1R and NK2R in the contractile activity of porcine inflammatory uterus. The macroscopic and histopathological changes of the uteri have been previously reported [22]. It has been disclosed that application of *E. coli* to the inside of the uterine horns caused an inflammatory exudate. As expected, the endometrium was swollen and red, and the blood vessels were clearly visible. The histopathological examination revealed the development of severe acute endometritis as determined by an increase in neutrophil counts and damage to the luminal epithelium and/or glands.

It is well known that NKRs are commonly found in peripheral tissues. They play a role in smooth muscle contraction and epithelial secretion [23]. The NK2R has been disclosed to mediate the contractility in rat uterus, while the NK1R may be involved in the contractile activity in mouse uterus [24]. In addition, the myometrium preparations obtained from pregnant women during cesarean section also revealed the effect of TKs on the uterine contractions. The order of contractile potency (NKA > SP) shown in this study suggests a stronger involvement of NK2R in human uterine contractility [24].

On the other hand, in pigs, NK1R was expressed at the RNA level not only in the uterus, but also in all parts of the female reproductive system [25]. The presented study showed the expression of NK1R and NK2R in the myometrium in the physiological state and as a result of inflammation. It was proved that the expression of proteins did not differ significantly in the CON and SAL groups, which indicates that the injection of physiological saline had no effect on the expression of these proteins. The bacteria intrauterine administration induced a significant increase in the content of NK1R and NK2R proteins in the myometrium in relation to the CON and SAL groups. This closely corresponds to the fact that TKs and their receptors are involved in the development of pathology. The nuclear factor kappa B (NF-κB) has been shown to be responsible for the regulation of NK1R expression during inflammation. Receptor transcription is induced, i.a. by interleukin (IL)-1β, IL-12 and IL-8 via NF-κB mediation [26,27]. It is therefore highly probable that the intrauterine injections of *E. coli* could also activate this mechanism. This is also suggested by a study by Li et al. [28] who revealed that activation of the extracellular signal-regulated kinase/NF-κB pathway contributes to the inflammatory changes induced by *E. coli* infection in the bovine endometrial tissue.

The present study showed that inflammation did not alter the distribution of the immunoreactivity of TK receptors in the myometrium. Expression of NK1R and NK2R in blood vessels and muscle cells of myometrium in all test animals may suggest that these cells are targets for TKs in both inflammatory and healthy tissues.

The expression of NK1R and NK2R in the myometrium obtained by Western Blot analysis as well as the presence of NK1R and NK2R in muscle myometrial cells found by immunohistochemistry suggest the participation of these receptors in the contractility of healthy and inflamed uteri. On the other hand, the expression of NK1R and NK2R in the blood vessels of all experimental groups indicates the role of SP and NKA in the vascular activity of myometrium. SP has a vasodilator effect on the vascularization of the uterus and ovaries. Additionally, TK-immunoreactive nerve fibers are present in tunica adventitia of large blood vessels in the female reproductive tract [29]. Published studies conducted on transversally cut strips of pig coronary artery confirmed the vasodilator effect of SP, NKA and NKB [30].

The present report described the role of SP, NKA and their receptors in uterine contractility. This is particularly important due to the fact that disturbances in uterine contractile activity contribute to the development of inflammation and its subsequent consequences [31]. Our previous studies have shown that the contractile amplitude and frequency in pigs are partly different between the myometrium and the endometrium/myometrium in response to active substances, e.g., galanin, ACh, somatostatin, neuropeptide Y and PGs [7,8,11,12,13,14,32]. Accordingly, the role of SP, NKA and their receptors was determined using two types of uterine strips. The viability and suitability of the material for research were checked in all the harvested uteri by incubation with ACh, which increased (CON, SAL groups) or decreased (*E. coli* group) the amplitude and enhanced the frequency (all groups) [11].

The presented study for the first time determined the participation of SP and NKA in the contractility of the porcine uterus, both in the physiological state and as a result of inflammation. The role of NK1R and NK2R in porcine uterine contractility has also not been studied. In both the CON and SAL groups, SP and NKA application caused a decrease in uterine amplitude and an increase in frequency compared to the time before these TKs administration. Only in one case was a statistically significant difference found between the two groups, and it concerned a higher frequency of endometrium/myometrium in the SAL group, than in the CON group after SP administration. Under physiological conditions, SP stimulates the contractility of the rat uterus [33] and causes strong contractions of fundus, corpus and antrum of canine stomach [34]. Additionally, the increase in contractility of the longitudinal muscle of the isolated guinea pig ileum in response to the SP was proven [35,36,37]. In turn, NKA induces contractions of isolated preparations of the uterine muscle of non-pregnant rats and mice [24].

Comparing the period before SP administration, in the *E. coli* group, the peptide decreased the amplitude as well as the frequency in both uterine strips. However, in this group, the amplitude was higher and the frequency was lower than in the CON and SAL groups. In turn, NKA in the *E. coli* group lowered the amplitude, but increased the frequency of both uterine strips compared to the period before NKA treatment. After using NKA the amplitude dropped, and the frequency enhanced in the *E. coli* groups versus other groups. It can be explained by the increase in uterine contractility-reducing substances accompanying the inflammation. It is well known that inflammatory cytokines such as tumor necrosis factor-α (TNF-α), IL-1β, IL-6 and IL-8 are common in the inflammatory uterus [38]. Bachar et al. [39] found that the TNF-α attenuated SP- and NKA-induced relaxation in a time- and concentration-dependent manner. The effect of pro-inflammatory substances on the reduction in SP-dependent uterine contractility may be confirmed by reports indicating the peptide expression reduction in pathological conditions. For example, it has been shown that the level of SP expression in duodenal nerves correlates with intestinal ulceration [40].

The published literature data indicate the involvement of SP and NKA on myometrial contractility via specific receptors. Analyzing the direction and level of significance of changes in response to NK1R and NK2R antagonists, we indicated that these receptors also participate in the uterine contractile activity in gilts from the CON, SAL and *E. coli* groups. In a healthy and inflamed uterus, NKA decreased the amplitude and increased the frequency, while the use of an elective NK2R antagonist had the opposite effect. This suggests that the peptide acts through this receptor. In the case of SP, in the CON and SAL groups there was a decrease in amplitude and an increase in frequency, while the use of a selective NK1R antagonist led to the assumption that the peptide exerts its effects through NK1R. In the *E. coli* group, the antagonist application did not change the amplitude and frequency. It is likely that in this case the inflammatory components were involved in regulating uterine contractility, which led to an increase in its reduction. An indirect effect of TKs on uterine contractility related to its action on the synthesis of compounds involved in modulating contractility cannot be excluded. Our previous reports indicate that ACh reduces contractility of the porcine uterus in inflammation [11]. SP and ACh are expressed in the motor neurons of the gut, and both are involved in smooth muscle contractility. SP has been shown to be a neurotransmitter involved in maintaining the muscle response to ACh [41]. It is therefore likely that SP mediates an ACh-modulated contractile response.

Analyzing the clinical aspect of the results revealed in the present study, it can be speculated that by the reduction in the inflammatory amplitude of the pig uterus, SP and NKA contributes to the accumulation of exudate inside the organ. Moreover, SP decreased, while NKA increased the frequency of inflamed uterus. Additionally, taking into account the fact that the NK2R antagonist abolished the inhibitory effect of NKA on uterine amplitude, it suggests the possibility of therapeutic use of the antagonist, as a drug increasing uterine contractility in inflammation. This can lead to an improvement in the effectiveness of reproductive disorders treatment as well as a reduction in economic losses on farms.

## 4. Materials and Methods

### 4.1. Animals and Experimental Procedures

All procedures were conducted according to Polish and EU regulations in the field of Animal Protection and Welfare guidelines and were approved by the Local Ethics Committee (Consent no. 65/2015). The methods of animal experimentation have been described in detail in our previous study [11]. Briefly, the experiment involved fifteen Large White/Landrace crossbred gilts (age 7–8 months, body weight/BW/90–120 kg). Gilts with no disruptions in reproduction such as discharges from vagina were admitted to the experiment. The second estrous cycle occurred regularly in all selected animals. The pigs obtained from a commercial fattening farm were kept at animal house (Faculty of Veterinary Medicine, University of Warmia and Mazury, Olsztyn, Poland) three days before the beginning of the study. All animals were housed in a standardized environment suitable to their species. The pigs were fed twice daily with a complete feed mix adapted to their needs and had access to water ad libitum.

Immediately after the assimilation period, the pigs were randomized on day 3 of the second estrous cycle (day 0 of the research) into three group: *E. coli*-(n = 5), saline-(SAL, n = 5) treated, or control (CON, n = 5) groups. In the *E. coli* group, the animals were intrauterine injected with *E. coli*, while in the gilts of SAL group, saline was administered also by intrauterine injections. The control gilts (CON group) were sham operated only.

The premedication was performed with atropine (0.05 mg/kg BW; Atropinum sulf. WZF, Warszawskie Zakłady Farmaceutyczne Polfa S.A., Poland), azaperone (2 mg/kg BW; Stresnil, Janssen Pharmaceutica, Beerse, Belgium) and ketamine hydrochloride (10 mg/kg BW; Ketamina, Biowet, Puławy, Poland). Ketamine hydrochloride was used for induction and maintenance of general anesthesia (supplementary doses—1 mg/kg BW every 5 min). In the next stage, a mid-line laparotomy was performed on pigs from the *E. coli* group for administration into each uterine horn 50 mL of *E. coli* suspension (strain O25:K23/a/:H1; Department of Microbiology, National Veterinary Research Institute, Puławy, Poland), containing 10^9^ colony-forming units/mL. In the SAL group, 50 mL of saline solution was administered. The bacterial suspension as well as saline solution were applied in 5 places (10 mL per each injection) at an identical distance from each other. After the injections, the uterine horns were massaged to distribute the solutions evenly. In the CON group, only a median laparotomy was carried out. In the period from surgery to euthanasia, all of the tested gilts were left untreated. Animals were euthanized by an overdose with the sodium pentobarbital on day 8 of the study, i.e., the expected day 11 of the estrous cycle. After confirming the cessation of biological functions, uterine tissues were collected for further analysis. For Western blotting, the fragments were collected from paraoviductal, middle and paracervical part of the uterine horn. With the aid of a scalpel blade and a dissecting microscope the endometrial and myometrial layers were separated. Next, the myometrium fragments (thickness of the entire layer) were immediately frozen in liquid nitrogen and then stored at −80 °C. To perform immunofluorescence staining, smaller pieces of uterine horn taken from three parts were placed in 4% paraformaldehyde solution (pH 7.4) for 24 h. After fixation, the tissues were rinsed in 0.1 M phosphate-buffered saline (PBS, pH 7.4). Subsequently, the uterine horn fragments were cryoprotected in an 18% buffered solution of sucrose (pH 7.4) until sectioning. To measure uterine contractility, the retrieved middle parts of the uterine horn fragments were placed on ice and immediately transported to the laboratory.

### 4.2. Western Blot Analysis

Following isolation, the myometrial tissues were homogenized on ice with a cold buffer composed of 50 mmol/L Tris-HCl, pH 8.0; 150 mmol/L NaCl; 1% Triton X-100, 10 mg/mL aprotinin, 52 mmol/L leupeptin, 1 mmol/L pepstatin A, 1 mmol/L EDTA, 1 mol/L PMSF and then centrifuged (2500× *g*, 4 °C, for 10 min). After centrifugation of the supernatants (17500× *g*, 4 °C, for 1 h), the collected material was stored at −80 °C. The Bradford method was followed to estimate the protein content [14]. Equal amount of protein (20 μg) was mixed with a sodium dodecyl sulfate (SDS) gel-loading buffer (50 mmol/L Tris-HCl, pH 6.8; 4% SDS, 20% glycerol and 2% β-mercaptoethanol), heated (95 °C, for 4 min), loaded and separated in 10% SDS-polyacrylamide gel electrophoresis. The separated proteins were then electro-blotted onto nitrocellulose membrane (0.22 μm) in transfer buffer. The composition of the transfer solution was 20 mmol/L Tris-HCl buffer, pH 8.2; 150 mmol/L glycine, 20% methanol and 0.05% SDS. Membranes were blocked with 5% nonfat dry milk in Tris-buffered saline containing 0.05% Tween-20 (0.05% TBS-T) (21 °C, for 1.5 h). Afterwards, membranes were incubated with rabbit polyclonal antibodies against NK1R (Novus Biologicals, Littleton, CO, USA, cat. no. NB 300-119, working dilution 1:500) and NK2R (LifeSpan BioSciences, Inc., Seattle, 161 WA, USA, cat. no. LS-C177106, working dilution 1:500) at 4 °C, for 18 h. In the next step, the membranes were washed in TBS-T buffer and then incubated (at 21 °C, for 1 h) with biotinylated goat anti-rabbit IgG (Vectastain Elite ABC-HRP Kit, Vector Labs, Burlingame, CA, USA, cat. no. PK-6101, working dilution 1:3000). To determine the visualization of antibody binding, incubation (for 3–4 min) with a mixture of 3,3′-diaminobenzidine tetrachloride (DAB, Sigma Aldrich, St. Louis, MO, USA, cat. no. D5637) and H_2_O_2_ in Tris-buffered saline (pH 7.2) was performed. In order to illustrate the specificity of the primary antibodies utilized, these were excluded from the analysis (negative control). A positive control was made with the mouse and porcine duodenal proteins. The glyceraldehyde-3-phosphate dehydrogenase was used to control for sample loading and protein transfer and to normalize the content of target protein. Signals were detected with a Quantity-One system (VersaDoc 4000 M imaging system, Bio-Rad Laboratories, Hercules, CA, USA).

### 4.3. Immunofluorescence

Fragments of uterine horns were embedded in OCT Tissue-Tek and cut at −22 °C into 10 μm thick sections using a cryostat (Reichert-Jung, Nußloch, Germany). The frozen sections were subjected to labeling using routine immunohistochemical staining according to method described previously [14]. In the next stage, the prepared sections were dried (at 21 °C, for 30 min) and then were washed in three changes of 0.1 M phosphate-buffered saline (PBS) for 15 min. Subsequently, preparations were incubated (at 21 °C, for 1 h) with buffered blocking mixture consisting of 0.1 M PBS, 10% normal goat serum (MP Biomedicals, Solon, OH, USA), 0.1% bovine serum albumin (Sigma-Aldrich, St. Louis, MO, USA), 0.05% Thimerosal (Sigma-Aldrich, St. Louis, MO, USA), 1% Triton X-100 (Sigma-Aldrich, St. Louis, MO, USA) and 0.01% sodium azide. After triple-rinsing in PBS (15 min), all sections were immunolabeled in humid chambers with primary antibodies (at 21 °C, for 18 h). At this stage of research, the same antibodies were used as in the Western blot method: NK1R (working dilution 1:200) and NK2R (working dilution 1:200). On the following day, sections were washed again in a PBS solution (3 × 15 min) and incubated with biotinylated anti-rabbit IgG (Chemicon International, Temecula, CA, USA, cat. no. AP132B, working dilution 1:1000) (at 21 °C, for 1 h), and next with carbocyanine 3 (CY3)-conjugated streptavidin (Jackson ImmunoResearch Labs, West Grove, PA, USA, cat. no. 016160084, working dilution 1:9000) (at 21 °C, for 1 h). Rabbit normal IgG was used to perform a negative control (instead of the primary antibodies). Positive control for NK1R and NK2R was obtained following the use the same antibodies as in the immunohistochemical staining. The porcine duodenal tissue was used to carry out the positive control. Stained sections were examined under a microscope with epi-fluorescence and appropriate filter sets for Cy3 fluorochrome (U-M41007) (Olympus BX51, Olympus Consilio sp. z o.o., Warsaw, Poland).

### 4.4. Preparation of the Strips from the Uteri and Measurement of Isometric Contractile Function

In the present study, two types of strips (3 × 5 mm) consisting of isolated myometrium and myometrium with associated endometrium obtained from fragments of the uterine wall were used to determine the contractile activity of uteri. The strips were then rinsed in saline and fixed between two stainless steel hooks in a 10 mL organ bath (Radnoti Unit Tissue Organ Bath System type 159920, Radnoti LTD, Dublin, Ireland) under 5 mN tension. Tissues were immersed in a Krebs-Ringer solution (pH 7.4) composed of (mM/L) NaCl, 120.3; KCl, 5.9; CaCl_2_, 2.5; MCl_2_, 1.2; NaHCO_3_, 15.5; glucose, 11.5. During the experiment, the solution was at a temperature of 37 °C and was constantly saturated with a mixture of 95% O_2_ and 5% CO_2_.

### 4.5. Contractility Study Schedule

**Figure 7:**
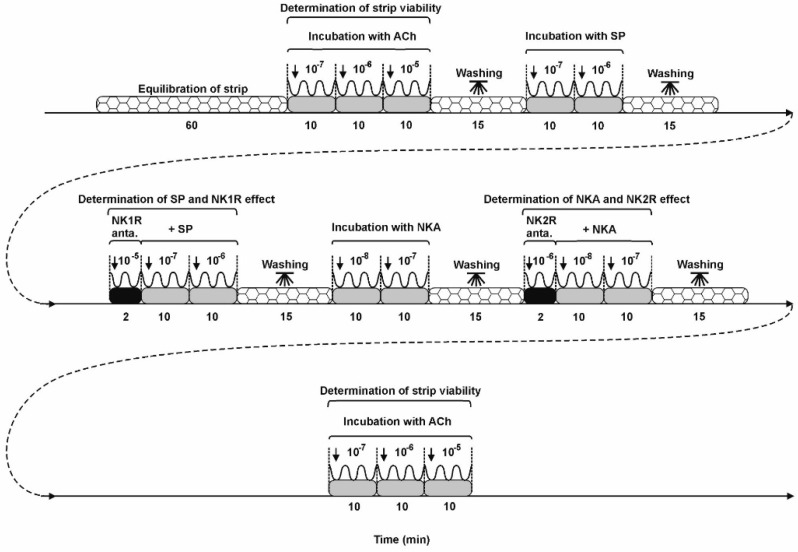
The graphic shows the procedure for measuring contractility of uterine strips. Ach-acetylcholine; SP-substance P; NKA-neurokinin A; NK1R-neurokinin receptor subtype 1; NK2R-neurokinin receptor subtype 2; NK1R anta.-neurokinin receptor subtype 1 antagonist; NK2R anta.-neurokinin receptor subtype 2 antagonist. Concentrations of the used substances are given in moles.

The exact procedure for handling uterine strips is illustrated in the diagram (Figure 7). Tissues were equilibrated and then spontaneous contractility of uterine strips was recorded for 1 h. Both amplitude (the difference between minimum and maximum values for a single contraction in mN) and frequency (the number of peaks) of the uterine contractions were determined using a force-displacement transducer and analyzed in a computer with PowerChart software (Chart v5, scope v5, ADInstruments, NSW, New South Wales, Australia). Initially, the strips were treated with ACh (Sigma, St. Louis, MO, USA, cat. no. A6625) at three dose strengths (10^−7^, 10^−6^, 10^−5^ M) to determine the viability of the uterine tissues and their suitability for the further steps of the experiment. In the next stage, the influence of SP (doses: 10^−7^, 10^−6^ M, Bachem, cat. no. H-1890) on the uterine contractility was noted. The action of each dose of SP was recorded for 10 min. It should be mentioned that uterine contractility was also determined in response to SP in the presence of NK1R antagonist (L-733,060 hydrochloride, TOCRIS, cat. no. 1145). For this purpose, uterine tissues were treated with the antagonist of NK1R (dose: 10^−5^ M, for 2 min) and then SP (doses: 10^−7^, 10^−6^ M) was applied. The antagonist and SP effects were evaluated for 10 min, then the sections were rinsed after each measurement (three times in PBS). In the next stage of the experiment, the effect of NKA on contractility of uterine strips was examined. After washing for 15 min, the tissues were exposed to NKA (TOCRIS, cat. no. 1152) at doses of 10^−8^ and 10^−7^ M recorded for 10 min. Then, as in the case of SP, the effect of NKA on uterine contractions in the presence of the antagonist of NK2R (TOCRIS, cat. no. 1274) was determined. First, the effect of the NKA2R antagonist (dose: 10^−6^ M, for 2 min) on uterine sections was determined and then NKA was added (doses: 10^−8^, 10^−7^ M, for 10 min). Similarly, the tissues were rinsed three times in PBS after each measurement. At the end of the experiment, tissue viability was re-checked using ACh according to the procedure described above. In the presented study, the results obtained from tissues in which the discrepancy in the response to ACh at the beginning and the end of the study did not exceed 20% were taken into account. The ACh concentrations used in this study have already been described in the available literature [8,32], while the concentrations of SP, NKA and receptor antagonists have been established in the preliminary studies. Using healthy porcine uteri and SP (doses: 10^−8^, 10^−7^, 10^−6^ M) alone and together with the NK1R antagonist (doses: 10^−6^, 10^−5^ M) as well as NKA (doses: 10^−9^, 10^−8^, 10^−7^ M) alone and together with the NK2R antagonist (doses: 10^−7^, 10^−6^ M) it was revealed that SP at doses of 10^−7^ and 10^−6^ M and NKA at doses of 10^−8^ and 10^−7^ M more effectively decreased the contractile amplitude. In turn, the antagonist of NK1R at a dose of 10^−5^ M and the antagonist of NK2R at a dose of 10^−6^ M statistically significantly the changed SP- or NKA-influenced, respectively, contractile amplitude.

### 4.6. Statistical Analyses

For each experimental group, the mean (±SEM) expression of NK1R and NK2R proteins was defined, and then the statistical significance was assessed using analysis of variance (ANOVA) with Bonferroni’s Multiple Comparison post hoc test (InStat Graph Pad, San Diego, CA, USA). The mean (±SEM) of amplitude and frequency were estimated for each group before administration of the substances and were taken as 100%. The effects of the peptides considered in this test are expressed as percentages (mean ± SEM) of these parameters determined prior to use. Statistical analysis was performed using the Bonferroni test to perform a contractility analysis between the results obtained before and after each administration of the active substances in each group as well as the mean values between the groups in response to the same added peptides. Differences were recognized significant at *p* < 0.05 (*), *p* ≤ 0.01 (**) and *p* ≤ 0.001 (***).

## 5. Conclusions

We have shown that endometritis conditions increased NK1R and NK2R protein expression in the porcine myometrium. The demonstrated involvement of the NK1R/SP and NK2R/NKA systems in the contractile function of an inflamed uterus suggests that these systems influence the severity of the inflammatory symptoms in the course of spontaneous endometritis/metritis. Based on these findings, the obtained results provide a better understanding of the pathomechanism of bacterial endometritis in animals as well as in humans, contributing to better prevention and treatment of uterine diseases.

## Data Availability

Data is contained within the article.

## Supplementary Materials

The following supporting information can be downloaded at: https://www.mdpi.com/article/10.3390/ijms232113184/s1.
