# Supplementary material for: Effects of Substance P and Neurokinin A on the Contractile Activity of Inflamed Porcine Uterus
Source: Int J Mol Sci. 2022 Oct 29;23(21):13184. doi: 10.3390/ijms232113184 (PMC9657707; doi:10.3390/ijms232113184)
Supplement: Supplementary file 1 [file ijms-23-13184-s001.zip › ijms-1984199-supplementary.pdf]

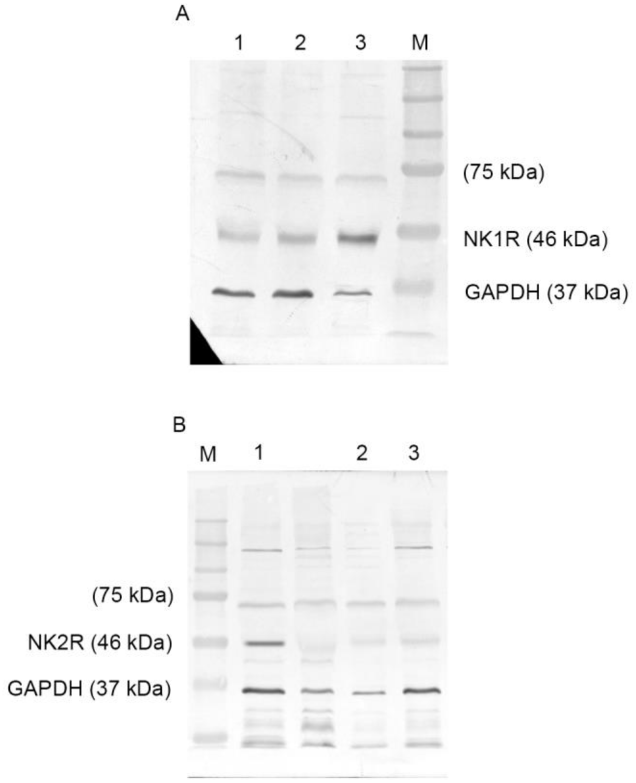

Figure S1. Western blots of NK1R and NK2R in the myometrial layer of gilts from the control (CON), saline (SAL) and *E. coli* groups. Panel A shows bands for NK1R in the CON (lane 1), SAL (lane 2) and *E. coli* (lane 3) groups. Panel B shows bands for NK2R in the *E. coli* (lane 1), CON (lane 2) and SAL (lane 3) groups. The density of bands was normalized in relation to glyceraldehyde-3-phosphate dehydrogenase (GAPDH). M—molecular weight markers.

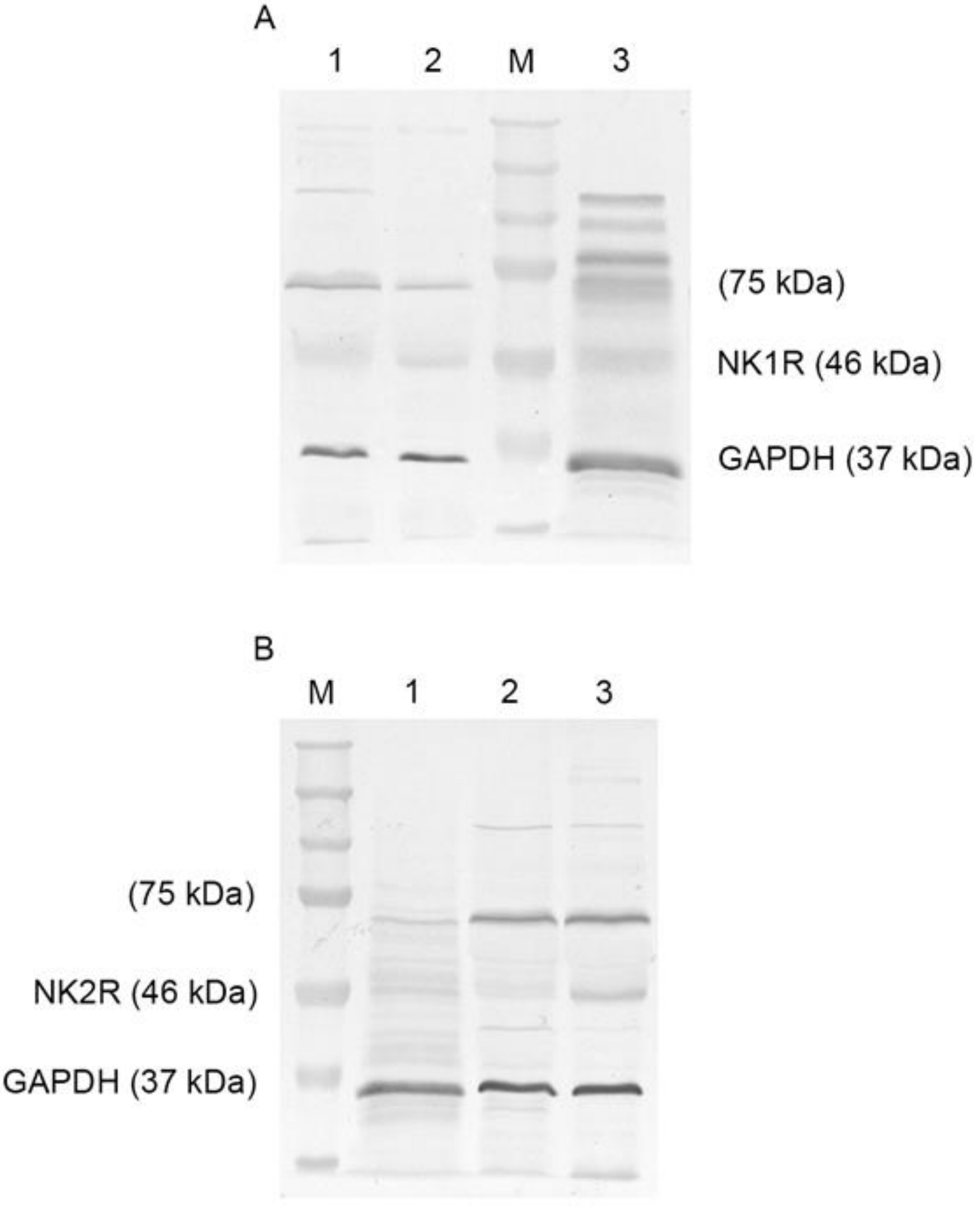

Figure S2. Western blots of NK1R and NK2R in porcine myometrium and relevant positive control. Panel A shows blots for NK1R antibody: specific bands at 46 kDa are visible in porcine duodenum (lane 1) and myometrium (lane 2), and mouse duodenum (lane 3). Panel B shows blots for NK2R antibody: specific bands at 46 kDa are visible in mouse (lane 1) and porcine (lane 2) duodenum, and porcine myometrium (lane 3). The density of bands was normalized in relation to glyceraldehyde-3-phosphate dehydrogenase (GAPDH). M—molecular weight markers.

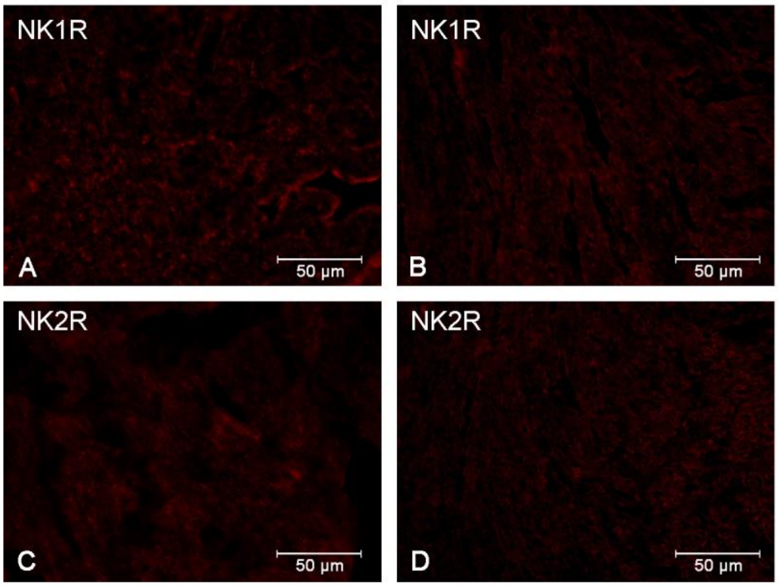

Figure S3. The immunoexpression of NK1R and NK2R in porcine myometrium and relevant positive control. The expression of NK1R is visible in porcine duodenum (A) and myometrium (B), and the expression of NK2R in porcine duodenum (C) and myometrium (D).
